# Does Perceived Green Space Quality Matter? Linking Norwegian Adult Perspectives on Perceived Quality to Motivation and Frequency of Visits

**DOI:** 10.3390/ijerph16132327

**Published:** 2019-07-01

**Authors:** Claudia Fongar, Geir Aamodt, Thomas B. Randrup, Ingjerd Solfjeld

**Affiliations:** 1 School of Landscape Architecture, Faculty of Landscape and Society, Norwegian University of Life Sciences, P.O. Box 5003, NO-1432 Ås, Norway; 2 Department of Public Health Science, Faculty of Landscape and Society, Norwegian University of Life Sciences, P.O. Box 5003, NO-1432 Ås, Norway; 3 Department of Landscape Architecture, Planning and Management, Swedish University of Agricultural Sciences, PO Box 58, 23053 Alnarp, Sweden

**Keywords:** perceived green space quality, Norwegian adult perspectives, motivation, activities, visit frequency

## Abstract

Individual perceptions of green space quality are essential when a user considers engaging in activities. This national-scale study provides insights into Norwegians’ quality perceptions of municipal green space, visit frequency and motivations for engaging in different activities. We applied regression analysis to investigate how various factors affect the outcome variables, quality perceptions and visit frequency from a sample of the Norwegian adult population. Results reveal that Norwegians perceive their green spaces as having good quality, and higher quality perceptions have a positive influence on green space visits. Half of the respondents visited green spaces out of intrinsic motives in high-quality environments providing fresh air, experiences of nature and quietness. It is essential, however, to take into account that less reported activity mirrors groups of respondents who least often visit green spaces.

## 1. Introduction

During the past 10–15 years, the demand for high-quality green space in areas close to where people live has increased due to the rise in individualisation, multi-cultural societies, and increased life expectancy [1]. Additionally, new public values [2] and an increasing acceptance of potentials that green spaces can provide for humans, have been in focus. Green spaces provide services such as the improvement of local climate, air quality [3], and carbon sequestration [4], along with supporting services such as biodiversity and habitat provision [5]. Studies have shown that human interactions with nature provide well-being and many desirable health outcomes [6,7], and overall policy visions aim to achieve well-being and quality of life through quality green space [8,9,10]. These needs are emphasised in agendas such as The New Urban Agenda, which was also endorsed by the United Nations General Assembly in December 2016. The United Nations calls for “promotion of safe, inclusive, accessible, green and quality public spaces… gardens and parks, …that are designed and managed to ensure human development and build peaceful, inclusive and participatory societies…” [11]. The new agenda on physical health launched in 2018, depicts green space as an integral part of delivering public health and quality of life [12]. Such policy guidance supports the emphasis put on green spaces and their role in providing quality of life and well-being for the population.

Moreover, regardless of the potentials that green spaces have, people need to consciously engage with green space to derive most of the benefits [13]. Thus, understanding how and why residents interact with green spaces nearby and how perceived quality influences public use of green space becomes increasingly important for managers and planners of such spaces [13,14,15].

A complex set of green space properties have been connected to increased green space visits [15]. The prevailing view is that the provision of clean and safe green spaces is particularly crucial for visiting green space [16,17,18,19,20]. Important factors that attract visitors to green spaces are cleanliness, naturalness, aesthetics, safety, access and appropriateness of development. Cleanliness is essential in any setting, although the characteristics of what defines cleanliness vary in the literature [18,21,22]. Other studies have explored specific features of green space deemed vital for attracting visitors [23,24,25]. Kaczynski and Havitz [25] examined the relationship between features in a park and residents’ physical activity, concluding that significant motivation for being physically active derives from a variety of features reflecting a range of reasons for using green space. Past research has also shown that, given the large diversity of features available, parks with more features are more likely to be used [16,23,26]. Various studies have investigated whether there is an association between people’s access to green space and the frequency of their visits. Studies typically use objective measurements such as distance to green space from the visitor’s home [27,28] or subjective proximity measures [15,29,30,31]. Moreover, researchers promote the use of subjective distance measures to predict visit frequency [1,30,31].

However, the relationship between motivation based on proximity and visit frequency may be moderated by quality perceptions of green space [32,33] and socio-personal characteristics [20,34]. Several studies have identified the effects of socio-personal characteristics on visit frequency and preferences for green spaces, including age [30,35], gender, and education [30]. However, these variables may not always have the same effect on visit frequency [36]. Green space is desired not only for its features; visits also depend on how the spaces are structurally patterned and, not least, on individual perceptions [37,38,39] and other users’ behaviour. Perceptions are found to be a stronger predictor for visits than objectively measured quantities of green space [13]. Such perceptions might prohibit or increase visits [40]. These perceptions modify the relationships between what is provided in terms of actual green spaces and what is perceived as quality green space by the public [27].

### 1.1. (Perceived) Quality Green Space

Public institutions manage green space and define what quality green space is. These organisations are differentiated, functional and operational closed systems in themselves. The production of quality green space is then a performance measured in its own code, and quality lies within the system. These systems sustain themselves, produce quality, create quality evaluations and tools to sustain their quality. So, the question is how the delivered quality is actually perceived as quality green space by the public?

In literature, quality has been defined through varying features that, combined, describe the quality or attractiveness of green space. Van Herzele and Wiedemann [38] illustrate the attractiveness of spaces through attributes that include spaciousness, nature, culture and history, quietness and facilities. Giles-Corti et al. [16] explored perceived quality attributes and activities in green spaces, using a composite index of park attractiveness (describing quality green space), incorporating environmental quality, three amenity factors and two safety factors as indicators. Ries et al. [33] measured perceived park quality through physical environment, social environment, organisational environment, and economic environment, which proved to be positively associated with park use. Bai et al. [41] explored perceived quality through seven quality items, including cleanliness and attractiveness.

Quality can be understood as both characteristics of a feature and as degree of excellence [42]. Characteristics of a feature describe the material it is made of and the condition, the quality of the property itself. On the other hand, the character of excellence is based on perceptions and experiences associated with the feature or the sum of features. Quality is hereby an overall impression of the excellence of the green space, describing green space character. Instead of measuring the quality given by indicators, perceived quality relies on respondents’ judgements of quality. Relying on the subjective preferences of the respondent, serving as an indicator of the excellence of green space character more than features of green space [43].

In this article, users’ overall quality perceptions of close-to-home green spaces are used to describe their judgement of the excellence of space, to judge the product of quality green space provided by public institutions. These perceptions are then related to motivation for all kinds of activities. Against the general focus on physical activities for example [44,45] this article considers all kind of activities, relying on the assumption that all outside activities, including visits to green spaces, have positive health effects.

### 1.2. Motivation for Activities

Little research in Norway has focused on the overall quality perceptions of green space and their relationship to proximity, visit frequency and motivation for activities. Nordic perspectives on activities within green space focus mainly on the relation of green space to health [44,46,47,48,49]. In Norway, activities in the outdoors are part of the national identity and culture [49]. However, eight out of 10 people live in urban areas [50], while just more than 2200 km^2^ (1.7%) of a total land area of 323,809 km^2^ is considered urban [51]. This means that natural environments are close to where people live, and 56% have safe access to recreational areas [52]. The very fact of being outdoors and the pure enjoyment of nature are motivations for visiting green spaces [48]. In a literature review, Calogiuri and Chroni [6] conclude that the quality of natural environments, especially safety, aesthetics and accessibility aspects, is essential for increasing physical activity in green spaces.

Hofmann [53] grouped activities carried out in green spaces according to their motivation and categorised experiencing nature as an intrinsic reason for visiting green space. Other motives include activities motivated by others (extrinsic), active motives or social motives [53]. As an explanation of motivational processes underlying the relationship of environment and activity, Ajzen’s theory of planned behaviour establishes that behaviour is mainly driven by intention. Motivation can be seen as the intention behind the execution of an activity, which in turn influences behaviour, i.e., the activity that is subsequently carried out [54].

Insights into motivation for users’ green space visits can enhance for the choices made in green space management so that the offer of green spaces coincides with user preferences. We assume that activities carried out in green spaces are optional activities. Optional activities take place when conditions for visiting a space are optimal. Such optimal conditions arise when green space features and perceptions of them create a quality green space for the individual and thus a space chosen for the given activity. It is essential, for example, to provide cleanness and safety because otherwise, optional activities would not be carried out. This means that the activities, and their related motives, can be used as an indicator for the quality of green space [55].

In this article, we aim to provide insights into Norwegians’ quality perceptions of municipal green space, visit frequency and motivations for engaging in different activities. The research questions of the study were, therefore: (RQ1) *Does perceived quality play a role in users’ visit frequency to green spaces?* and (RQ2) *How does the quality of green space relate to users’ motivation for activities?*

## 2. Materials and Methods

### 2.1. The Survey

The survey was administered via telephone by an external and independent market research company (Norstat) during January 2018. The sample in the study comprised 1010 Norwegians 18 years of age or older. This sample, which was stratified by age, gender and geography, was drawn from Norstat’s permanent panel of respondents that are surveyed every week and are representing the Norwegian population.

### 2.2. Survey Questions

The questionnaire was grouped into five sets of questions; quality of green space, activities, visit frequency, distance to green space, and a set of socio-demographic variables.

The first questions aimed to assess the perceived quality of municipal green spaces in general; how do you perceive the quality of green spaces in your municipality? The five answer options ranged from very bad quality to very good quality, allowing for the overall judgement of the quality of green spaces provided by public institutions.

The second question invited the respondents to focus on a green space they visited most during summer, between April to October. With a focus on the specific green space, respondents were asked to list the activities they performed (Table 1). Table 1 presents the four groups of motivations for activities; (1) extrinsically motivated activities, (2) activities motivated by social interaction, (3) active motivation, and lastly, (4) intrinsically motivated activities.

We focused on activities that can be carried out during warmer temperatures, inspired from [30], and kept our focus on three to five activities for each motivation group. Respondents could choose as many activities as they wanted. A fourth category ‘*do not visit green space*’ was added to account for non-users. If this option was chosen, the respondents got no further questions about their engagement with green spaces.

Respondents were then asked to state the frequency of visits in the same timeframe. The answer options were daily, several times per week, weekly, monthly and less than monthly based on [45,56]. In the last questions, respondents were asked to report how far the green space was from their home. Answer options included six distances: less than 50 m, 50–149 m, 150–299 m, 300–999 m, 1–5 km or more than 5 km based on [31,46,57].

### 2.3. Statistical Analysis

All data analysis was carried out using R [58]. Firstly, we conducted a descriptive analysis of the sample, using Pearson Chi-square (χ^2^) tests, to study the associations between categorical variables, quality perception and visit frequency and predictor variables. Potential predictors included in the analysis were sociodemographic characteristics of the respondents. Age was grouped into six groups (18–29, 30–39, 40–49, 50–59, 60+ years). Educational level was split into two categories: those with ground and secondary education (≤12 years) and those with higher education (including university) (>12 years). Household income was separated into three groups; below average household income (estimated at 600,000 NOK), between 600,000 and 1 million NOK and more than 1 million NOK (approx. 10 NOK = 1 EURO). The presence of children under 18 years of age in the household was operationalised as a binary variable (None/more). The degree of urbanisation was divided into three categories: urban (Oslo (the capital, 681,067 inhabitants) and cities more than 50,000 inhabitants), suburban (cities with between 5000 and 50,000 inhabitants) and rural (towns with less than 5000 inhabitants). Regions in Norway were merged into four regions according to geography, and population size: Northern and Central Norway, Eastern-Norway, Oslo, and Western and Southern Norway.

Secondly, we fitted two linear regression models. We used a stepwise forward variable-selection procedure (R Mass Package) to find the best model to explain visit frequency and quality perception. The results of the Pearson Chi-square test run in the first step determined hereby the predictor variables included. Confidence intervals were calculated using a profile likelihood method [59]. A measure of explained variance for the model was reported as (*R*^2^).

Lastly, to investigate the individual activities, which were dichotomous, we fitted logistic regression models for each activity. We used a stepwise backward variable-selection procedure to find the best model explaining the association between perceived quality and the predictor variables. *p*-values less than 0.05 were considered statistically significant. We evaluated each model using the Akaike’s information criterion, which is suitable for determining the trade-offs between the goodness-of-fit and the complexity of the model. Some predictors reduced the Akaike’s information criterion for some activities, and we report the best fit model.

## 3. Results

### 3.1. Population Characteristics

The characteristics of the study’s population sample and the variables used in the study are listed in Table 2. The sample was balanced with respect to gender (51% females and 49% males) and age. Most of the respondents had no responsibility for small children (72%), they lived in urban areas (38.7%), and within 1-km distance to their most visited green space (59.1%). Almost 70.0% of the respondents perceived the overall quality of their municipality green spaces as good (69.7% female; 68.3% male). In relation to national figures, our sample was well balanced with respect to age, gender, the degree of urbanisation and households with children under 18, although the group with higher education appeared to be somewhat overrepresented [50,60,61].

### 3.2. Predictors for Visit Frequency and Quality

In Table 3, we report the relationship of predictors to green space quality and visit frequency. We found that participants living less than 300 m from a green space assessed the quality of their nearest green space higher than those who stated that they have between 300 m and 5 km to their nearest green space (*p* < 0.001). Participants living in eastern Norway assessed the quality of their nearest green space higher than participants living in Northern and Central Norway (*p* = 0.004). Individuals who visited green spaces less than once a month assessed the quality as being lower, compared to individuals visiting green areas more than once a week (*p* < 0.001). The explained variability of quality assessment was 5.3%.

Distance to green space was the strongest predictor for visit frequency (*p* < 0.001) (Table 3), with visit frequency significantly decreasing for distances of more than 300 m and even more when the green space is more than 5 km away. High level of education was also significantly associated with visit frequency; respondents with higher education visited green spaces more frequently than those who had a lower education (*p* = 0.003). Green space quality was significantly associated with visit frequency; individuals rating their nearest green space as ‘bad’ visited green space 0.319 times less than those rating their green space as ‘good’ (*p* = 0.024). The explained variability of visit frequency was 14.3%.

### 3.3. Predictors for Activities

**Figure 1:**
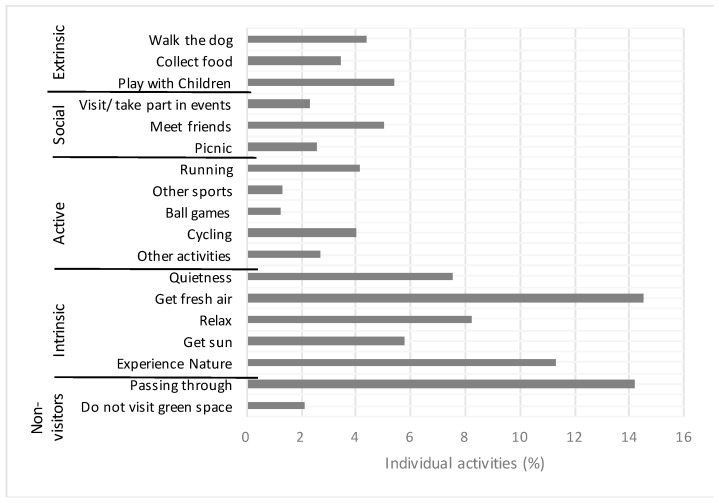
Percentage of activities for the different groups of motivating factors based on a study of 1010 adult Norwegians.

An overview of the reported activities is shown in Figure 1. Among the intrinsically motivated activities, adult Norwegians visit green space mostly to get fresh air (14.5%), experiencing nature (11.3%) or visiting green space to relax (8.2%). Extrinsic motivation is mentioned by 13.2% of the respondents, visit/take part in events (2.3%), collect food (3.5%), and play with children (5.4%). Activities motivated by action are mentioned by 13.4% of the respondents. Respondents choosing to pass through green spaces account for 14.2%, while 2.1% do not visit green spaces at all.

Different predictors were associated with different activities; patterns were nonetheless identifiable, and the outcomes of the individual linear regression modelling are presented in Table 4.

Quality is a strong predictor for intrinsically motivated activities (see Table 4). Positive quality perceptions increase visit frequency. Besides, getting fresh air, experiencing nature and visiting green spaces for relaxation are more likely to be motivations of female respondents. The degree of urbanisation was significant for relaxation, where inhabitants living in urban areas were more likely to visit green spaces for relaxation. However, experiencing nature as a motivation for visiting green spaces was less likely for urban inhabitants.

Extrinsically motivated activities are significantly related to gender (see Table 4). Female respondents are more likely to visit green spaces for activities, such as playing with children, collecting food and walking the dog. Distance plays a crucial role for walking the dog and activities with children, while a greater distance to green spaces reduces visit frequency for extrinsic motivations.

Significant predictors for active-motivated activities are gender, distance and education (see Table 4). Males are more likely to engage in active activities, running, cycling and engaging in ball games. Distance to green spaces decreases all types of active motivated activities. Higher education corresponds with increased visits for running and cycling activities; however, ball game activities decrease with higher education.

Socially motivated activities are significantly related to age (see Table 4). Commonly, engaging in social activities decreases with increasing age. Yet, a pattern for picnicking emerges, age groups of 30–39 are more likely to visit a green space for a picnic than the age groups 18–29.

A commonly shared trait of respondents who do not visit green space is their quality perception (see Table 4). Spaces considered of average or poor quality are visited less frequently. Additionally, there are significantly fewer non-visitors in urban and suburban areas than in rural areas.

## 4. Discussion

To the best of the author’s knowledge, the present study was the first to investigate the relationship between perceived measures of quality, visit frequency, and motivation for activities in Norway. Perceived measures, even if they are not in correspondence with objective measures, are essential since perceptions are the basis for individual decisions. We asked respondents specifically about the green space they visit most frequently. We assumed that these green spaces are close to their homes, irrespective of the type of green space. Such spaces are used more frequently and are visited most of the time because of their proximity, rather than the individual’s attraction to or fascination with the green space. Thus, respondents may have an immediate relationship with a green space close to their home [41].

In our study, respondents give a judgement on how they perceive the overall quality of their most visited green space provided for them by public institutions. By doing this, green space can be judged related to an overall impression instead of valuing features of space, as quality or attractiveness indicators tend to do. This judgement might be seen as superficial; however, preferences and socio-personal characteristics are considered, giving respondents the opportunity to evaluate the organisation that produces green space quality.

### 4.1. (RQ1) Does Perceived Quality Play a Role in Users’ Visit Frequency to Green Spaces?

The results demonstrated that visit frequency is related to age, level of education, distance to and quality perceptions of green space.

The strongest predictor of whether Norwegians will visit green space is self-reported distance. When their homes are more than 300 m away from a green space, the number of visits decreases. Earlier studies confirm this finding. Flowers et al. [32] investigated the relationship of subjective predictors of visit frequency within a UK nation-wide survey, showing that 67.7% of participants visit green space close to their homes at least a few times a month. Proximate parks encouraged park use in Perth, Australia [16], and access was identified as a defining factor for park visits in five Southeast European cities [36].

Having a higher education indicates increased visits to green spaces in our study; this tendency, however, is contrary to findings of a study in Denmark, where education had no relation to the frequency of use [30].

We found that positive quality perceptions were related to an increased number of visits. Quality green space thus provides optimal conditions that allow for activities to happen. Other literature refers to essential green space properties that provide such conditions. McCormack et al. [18] found that quality measures such as lack of maintenance influenced park use, especially dirty un-kept areas, the presence of litter and overfull rubbish bins were mentioned. Similar, Ostoić et al. [36] found that the lack of waste bins, signs of vandalism and litter were important issues preventing green spaces visits. Appropriate maintenance is perceived as highly significant and poor maintenance evokes negative perceptions of green space [36,45].

### 4.2. (RQ2) Does Quality Relate to Users’ Motivation for Activities?

Almost half of the sample of Norwegians are motivated by intrinsic reasons to visit green spaces. Norwegians have a close connection to nature, and the pure enjoyment of being outside and experiencing nature is an important motive. In a historical perspective, Norwegian recreation was often associated with quietness and solitude [62]. Similar, Calogiuri and Elliott [44] found experiencing nature to be the second most important motive for engaging in activities. Calogiuri et al. [47] found that fresh air is frequently reported by Norwegians when asked to describe nature experiences. Additionally, Hervik and Skille [48] found that fresh air was found to be mentally cleansing in their interview study of middle-aged and elderly laymen living in rural towns in Norway. Intrinsically motivated activities are strongly associated with positive quality perceptions. Motives for visits such as getting fresh air, experiencing nature and quietness indicate preferences for quality green spaces of a natural character. Natural environments are diverse, besides providing fresh air, green spaces preserve habitat and enhance biodiversity, indicating the relationship of activities carried out, and the services green spaces provide. The relation between intrinsic motivation and quality green spaces suggests that visits are primarily carried out in a space of high quality, where quality relates to nature, with vegetation and trees that absorb pollutants, reduce noise and thus provide fresh air. Besides positive quality perceptions, female respondents were more driven by intrinsic motivated activities than men (except for the motivation of experiencing nature, where we did not find any gender-related differences). This is confirmed in the research of Calogiuri and Elliott [44], where females were found to rate the importance of motives generally higher than males. This might indicate that women appreciate aesthetic and well-being values more highly than men do [63]. Getting fresh air and experiencing nature are both activities significantly associated with higher education, which was similar to relations found by Ostoić et al. [36], who found that the more highly assessed importance of urban forests was related to higher education.

We found several differences between Norwegian women and men in engaging in activities. Norwegian women were more motivated by extrinsic activities than men. Women were also significantly more likely to walk the dog, collect food items and play with their children. Our results showed that walking the dog and playing with children was strongly associated with distance to green space. Similar, Gundersen [62] reported that Norwegian children’s use of green areas was strongly associated with increasing distance to nature, as a 100-m distance from green places to their homes meant decreased use. On the contrary, collecting food is related to higher perceived quality of the green space. Green spaces have a certain quality that provides optimal conditions, for example, for picking mushrooms and berries.

Norwegian men are more active than females. Male respondents are more likely to engage in running, cycling and ball games. These activities decrease with greater distance to green space (over 5 km). Running and cycling were also significantly associated with quality perceptions of green space. Additionally, education relates to increased visits for running and cycling activities, which is also reported by Schipperijn et al. [56], where higher education was found to be significantly associated with outdoor physical activity in the nearest green space. However, bad quality perceptions as opposed to good quality perceptions did not influence running, which might indicate that active Norwegians run despite the conditions. Engaging in ball games was also not influenced by quality perceptions.

Norwegians’ social motivation—taking part in events, meeting friends and visiting green spaces for picnics—is strongly associated with age. Increasing age decreases visits for socially motivated activities. None of the socially motivated activities were related to quality perception, and except to take part in events, none were related to distance to green space either. This indicates that a greater attraction for events exists than for the actual character of the space. Visiting events might relate to the emergence of new concepts such as urban farming, agricultural initiatives [64] or outdoor sports arrangements and exhibitions, creating social arenas which may attract young people to urban forests and green spaces [62]. Such events might be visited more often if such events took place in a space close to our homes.

Norwegians use green spaces as a transitional passage from one place to get to somewhere else. Even though passing is not an activity, passing a green space indicates that people walk through green space as a deliberate alternative to street environments. The largest number of people walking is found in distances of less than 1 km, and the use of cars to drive to visit green spaces is higher for longer distances. This also means that an essential criterion for walking is found within the city structure where people live and work [65]. Passing through a green space is significantly related to urban and suburban areas, which is logically related to shorter distances to points of need (schools, kindergartens, shops). Norwegian governmental planning guidelines in 2014 emphasised that the increase in urban transport should be absorbed by public transport, cycling and walking [66]. Our results point to the potential creation of such better networks of green spaces that people utilise in urban areas. Only a small percentage of Norwegians do not visit green space, and non-visitors primarily perceive green spaces as having bad quality.

## 5. Strengths and Limitations

To the best of the author’s knowledge, the present study was the first to investigate the relationship between perceived measures of quality and visit frequency, and motivation for activities in Norway in a large national sample.

The interviewers of the research company followed a strict order of questions and answer options. We assumed that there might be a bias on the part of the respondents to first-mentioned activities and launched a control survey with randomly ordered answer categories for activities to control for this bias. The control survey was administered online via Questback and then announced on the university Facebook page, showing similar results for activities as our original survey.

Our definition of green spaces was quite open and could be interpreted in different ways. In general, there is no universally accepted definition of green space, and Norwegian municipalities define spaces differently. This might have led to an over- or under-estimation of green space visits. This also limits the understanding of activities in specific green spaces. In addition, respondents were only given a choice of 18 activities, whereas other activities may also be important for encouraging green space visits, especially winter activities, and these have not been considered.

Besides, the time of the year when the survey was conducted (October 2017 to December 2017) was not optimal, since respondents had to remember how they perceived green space quality during the summer months (April to October). Other research spent summer months to do their research on outdoor activities [15]; this was not possible for our survey.

We used a set of predictor variables to identify patterns within the Norwegian population sample to study how quality was related to different activities. Ideally, more variables should be included to better understand these associations, such as the participants’ ethnicity, profession, and level of physical activity. This is a cross-sectional study and causality cannot be inferred from the observed relationships. We do not think selection bias is a problem in our study. The study also focused on associations, where we compared groups to a greater extent than we estimated prevalence.

## 6. Conclusions

Understanding how and why residents interact with green space becomes increasingly important for the management and planning of nearby green spaces so that green spaces can fulfil their role in preserving and enhancing residents’ quality of life. The present study fulfilled two goals. Firstly, it discovered the role of Norwegians’ overall quality perceptions of and visit frequency to green spaces provided by public institutions. Secondly, it revealed the relation between activities carried out and visitors’ characteristics, quality, visit frequency and distance on a national scale.

Overall quality perceptions can indicate preferences of users for green spaces provided by public institutions. By using this information, green space quality provided can be judged, and managing as well as planning priorities might be set.

Norwegians perceive their green spaces as being of good quality, and higher quality perceptions influence green space visits positively. The strongest predictor for visits is perceived distance; spaces close to home are visited more frequently than faraway spaces. At the same time, those perceiving their neighbourhood green space quality as bad visit them less frequently than those perceiving their green spaces as good. Many Norwegians pass through green space, especially in urban and suburban areas. This indicates that a better network of spaces exists in more urbanised areas and that Norwegians consciously choose alternative routes in order to pass through green spaces.

Half of the Norwegians visited green spaces out of intrinsic motives. Intrinsically motivated activities are carried out in high-quality environments, indicating a conscious decision to engage in an activity in a nature-like environment of high quality. An increase in green space visits contributes to inhabitants’ quality of life. Therefore, it seems advisable to provide quality green space that facilitates intrinsically motivated activities, meaning nature-like environments that provide a space for quietness and contemplation, breathing fresh air and the possibility to experience nature’s varieties. To facilitate intrinsic reasons for visiting green space in strategic and operational goals for management and planning might enhance visitation. Moreover, from a public health perspective, green spaces and their services are vital to increasing health and well-being. Visiting spaces to get fresh air, to experience nature, to run and cycle is related to higher education. Educational campaigns might be used to increase awareness of the benefits of green spaces and thus positively affect human behaviour.

However, from a planning perspective, it is essential to consider that less-reported activity mirrors groups of respondents who visit green spaces the least. Green space features permit different activities for different groups, and spaces close to home with play equipment are vital for Norwegians with children. In our study, perceived overall quality did not predict the frequency of playing with children and walking the dog. One explanation is that playing with children and walking the dog are carried out despite the nature of green space. On the other hand, quality spaces close to home provide opportunities to run and cycle. The nature of such spaces most likely includes appropriately maintained paths, connectivity within a space and towards other spaces, appropriate lighting and other facilities necessary to carry out these activities. Equally important is the fact that events that are close to home increase visits; with this in mind, local initiatives that engage inhabitants and invite them to visit green spaces might increase visits. Subsequently, it is vital to keep and increase the establishment of green spaces close to where people live to engage everyone in an active lifestyle.

With this in mind, specific tools and measure, as described in the literature, seem necessary to indicate quality of features and to keep quality within the individual green spaces. As indicated by our results and based on the different green space quality perceptions, it seems plausible to use measures for quality that include users’ preferences, such as the Nordic Green Space Award [2] or similar measures where the overall character of a green space is judged, but also qualitative statements and revisions of the management are made.

## Figures and Tables

**Table 1:** Motivation and activities.

Motivation	Activity Categories
Extrinsic	Walk the dog; Collect food; Play with children
Social interaction	Visit/take part in events; Meet friends; Picnic
Active	Running; Other sports; Cycling; Ball games; Other activities
Intrinsic	Quietness; Get fresh air; Relax; Get sun; Experience nature
Non-Users	Passing through; Do not visit green space

**Table 2:** Population characteristics and Pearson chi-square test (χ^2^) results for quality and visit frequency and predictor variables derived from a Norwegian study of 1010 adults (significance levels: 0 ‘***’, 0.001 ‘**’, 0.01 ‘*’).

Variable	Total (%)	perceived quality (χ^2^)	Visit frequency (χ^2^)
N	1010		
Gender		0.219	0.289
Male	49.0		
Female	51.0		
Age		0.598	0.911
18–29	15.9		
30–39	17.9		
40–49	19.7		
50–59	12.9		
60+	33.6		
Education		0.001 ***	0.002 **
Lower	39.1		
Higher	60.9		
Yearly household income		0.75	0.116
Below average	31.4		
Above average	31.7		
More	36.9		
Household with children U18		0.564	0.812
None	72.0		
One or more	28.0		
Degree of Urbanisation		0 ***	0.585
Urban (>50,000)	38.7		
Suburban (5000–50,000)	31.2		
Rural (<5000)	30.1		
Region		0.005 **	0.42
Oslo	12.1		
Northern and Central Norway	23.4		
Eastern Norway	36.4		
Western-and Southern Norway	28.1		
Distance	(n = 936)	0.004 **	<0.001 ***
<300 m	40.6		
300 m–5 km	50.5		
>5 km	7.9		
Frequency	(n = 936)	<0.001 ***	-
Several times a week	31.7		
weekly	34.7		
less	33.5		
Quality	(n = 970)	-	<0.001 ***
Good	68.1		
Average	24.9		
Bad	6.9		

**Table 3:** Linear regression model outcomes with stepwise backward inclusion of significant predictor variables for visit frequency and quality perception based on a sample of 1010 adult Norwegians. *p*-value significance levels: 0 ‘***’, 0.001 ‘**’, 0.01 ‘*’, 0.05 slopes and 95% confidence intervals.

Variables		Perceived quality			Visit frequency		
		*p*	Slope	97.5% CI	*p*	Slope	97.5% CI
Perceived quality	Good				0 (Ref)	0 (Ref)	0 (Ref)
	Average				0.075	−0.139	(−0.293–0.014)
	Bad				0.024 *	−0.319	(−0.596–0.414)
Frequency	Several times a week	0 (Ref)	0 (Ref)	0 (Ref)			
	Weekly	0.311	−0.213	(−0.203–0.064)			
	Less	3.37 × 10^−5^ ***	−0.17	(−0.441–(−0.159))			
Education	Lower				0 (Ref)	0 (Ref)	0 (Ref)
	Higher				0.003 **	0.211	(0.074–0.349)
Region	Northern- and Central Norway	0 (Ref)	0 (Ref)	0 (Ref)			
	Oslo	0.298	0.101	(−0.09–0.292)			
	Eastern Norway	0.004 **	0.212	(0.068–0.357)			
	Western-and Southern Norway	0.065	0.143	(−0.009–0.295)			
Distance	<300 m	0 (Ref)	0 (Ref)	0 (Ref)	0 (Ref)	0 (Ref)	0 (Ref)
	300 m–5 km	<0.001 ***	−0.213	(−0.330–(−0.096)	6.36 × 10^−13^ ***	−0.509	(−0.646–(−0.372))
	>5 km	0.112	−0.17	(−0.38–0.038)	<2 × 10^−16^ ***	−1.068	(−1.316–(−0.82))
R^2^ (%)		5.3			14.3		

**Table 4:** Linear regression model outcomes with stepwise backward inclusion of significant predictor variables for activities based on a sample of 1010 adult Norwegians. Presented as slope values and *p*-value significance levels: 0 ‘***’, 0.001 ‘**’, 0.01 ‘*’, 0.05.

		Intrinsic				Extrinsic			Social			Active			Non-visitors	
		Quietness	Get Fresh Air	Relax	Experience Nature	Walk the Dog	Food Collection	Play with Children	Visit/Take Part in Events	Meet Friends	Picnic	Running	Cycling	Ball Games	Passing	Do Not Visit
	n	263	509	288	397	153	121	189	81	175	89	145	140	43	497	74
Perceived quality	Good	0 (Ref)	0 (Ref)	0 (Ref)	0 (Ref)		0 (Ref)	-	-	-	-	0 (Ref)	0 (Ref)	-	-	0 (Ref)
	Average	−0.398 *	−0.728 ***	−0.356	−0.941 ***		−0.836 **	-	-	-	-	−0.705 **	0.069	-	-	0.784 *
	Bad	−0.291	−0.796 **	−0.796 *	−1.05 **		−0.083	-	-	-	-	0.195	−1.376	-	-	1.632 ***
Distance	<300 m	-	-	0 (Ref)	0 (Ref)	0 (Ref)	-	0 (Ref)	0 (Ref)	-	-	0 (Ref)	0 (Ref)	0 (Ref)	0 (Ref)	-
	300 m–5 km	-	-	−0.195	−0.21	−0.428 *	-	−0.447 *	−0.702 **	-	-	−0.08	0.135	−1.117 **	0.28	-
	>5 km	-	-	−1.003 **	−0.931 **	−0.671	-	-0.735	0.026	-	-	−1.849 *	−1.448 *	0.268	0.39	-
Gender	Female	0 (Ref)	0 (Ref)	0 (Ref)	-	0 (Ref)	0 (Ref)	0 (Ref)	-	-	0 (Ref)	0 (Ref)	0 (Ref)	0 (Ref)	0 (Ref)	-
	Male	−0.345 *	−0.33 ***	−0.328 *	-	−0.392 *	−0.784 ***	−0.532 **	-	-	−0.418	0.524 **	0.382 *	1.538 ***	0.442 **	-
Age	18–29	-	0 (Ref)	0 (Ref)		0 (Ref)	-	0 (Ref)	0 (Ref)	0 (Ref)	0 (Ref)	0 (Ref)	-	0 (Ref)	-	-
	30–39	-	−0.181	−0.167	-	−0.088	-	1.442 ***	−0.772 *	−0.79**	0.894 **	−0.631 *	-	−2.135 ***	-	-
	40–49	-	−0.427	−0.609 *	-	0.275	-	0.503	−1.239 **	−1.601 ***	−0.215	−0.973 **	-	−0.907	-	-
	50–59	-	−0.549 *	−0.69 *	-	0.478	-	0.73	−1247 **	−1.742 ***	−0.919	−1.137 **	-	−2.145 **	-	-
	60+	-	−0.103	−0.555 *	-	−0.451	-	1.305 ***	−1.933 ***	−1.527 ***	−1.287 **	−2.403 ***	-	-	-	-
Education	Lower	-	0 (Ref)	-	0 (Ref)	-	0 (Ref)	-	-	-	-	0 (Ref)	0 (Ref)	0 (Ref)	0 (Ref)	-
	Higher	-	0.256	-	0.22	-	0.697 **	-	-	-	-	0.582 *	0.405	−0.698	−0.345 *	-
Children <18	None	0 (Ref)	-	-	-	-	-	0 (Ref)	0 (Ref)	-	-	-	-	0 (Ref)	0 (Ref)	-
	One	−0.349 *	-	-	-	-	-	2.294 ***	0.482	-	-	-	-	1.197 **	−0.44 **	-
Degree of urbanisation	Rural	0 (Ref)	-	0 (Ref)	0 (Ref)	-	0 (Ref)	-	-	-	-	0 (Ref)	-	-	0 (Ref)	0 (Ref)
	Suburban	−0.526 **	-	−0.179	−0.56 5**	-	−0.663 **	-	-	-	-	0.636 *	-	-	0.449 *	−1.087 **
	Urban	0.124	-	0.514 **	−0.597 ***	-	−1.1 ***	-	-	-	-	0.777 **	-	-	0.926 ***	−1.394 **

## Supplementary Materials

Supplementary File 1
