# Supplementary material for: Does Perceived Green Space Quality Matter? Linking Norwegian Adult Perspectives on Perceived Quality to Motivation and Frequency of Visits
Source: Int J Environ Res Public Health. 2019 Jul 1;16(13):2327. doi: 10.3390/ijerph16132327 (PMC6651101; doi:10.3390/ijerph16132327)
Supplement: Supplementary File 1 [file ijerph-16-02327-s001.zip › Figures_Fongar.docx]

Figure 1: Percentage of activities for the different groups of motivating factors based on a study of 1010 adult Norwegians.

Table 1: Motivation and activities *(based on Hofmann, 2011)*

| Motivation | Activity categories |
| --- | --- |
| Extrinsic | Walk the dog; Collect food; Play with children |
| Social interaction | Visit/ take part in events; Meet friends; Picnic |
| Active | Running; Other sports; Cycling; Ball games; Other activities |
| Intrinsic | Quietness; Get fresh air; Relax; Get sun; Experience nature |
| None-Users | Passing through; Do not visit green space |

*Table 2:* *Population characteristics and Pearson chi-square test (χ2) results for quality and visit frequency and predictor variables derived from a Norwegian study of 1010 adults (significance levels: 0 ‘***’, 0.001 ’**’, 0.01 ’ *’)*

| Variable | Total (%) | Perceived quality (χ2) | Visit frequency (χ2) |
| --- | --- | --- | --- |
| N | 1010 |  |  |
| Gender  Male  Female | 49.0  51.0 | 0.219 | 0.289 |
| Age  18-29  30-39  40-49  50-59  60+ | 15.9  17.9  19.7  12.9  33.6 | 0.598 | 0.911 |
| Education  Lower  Higher | 39.1  60.9 | 0.001 *** | 0.002 ** |
| Yearly household income  Below average  Above average  More | 31.4  31.7  36.9 | 0.75 | 0.116 |
| Household with children U18  None  One or more | 72.0  28.0 | 0.564 | 0.812 |
| Degree of Urbanisation  Urban (> 50,000)  Suburban (5,000 – 50,000)  Rural (< 5,000) | 38.7  31.2  30.1 | 0 *** | 0.585 |
| Region  Oslo  Northern and Central Norway  Eastern Norway  Western- and Southern Norway | 12.1  23.4  36.4  28.1 | 0.005 ** | 0.42 |
| Distance  < 300m  300m – 5km  > 5km | (N=936)  40.6  50.5  7.9 | 0.004 ** | <0.001 *** |
| Frequency  Several times a week  weekly  less | (N=936)  31.7  34.7  33.5 | <0.001 *** | - |
| Quality  Good  Average  Bad | (N=970)  68.1  24.9  6.9 | - | <0.001 *** |

The reduced sample size is due to the exclusion of respondents who answered that they “do not visit” green space

Table 3: *Linear regression model outcomes with stepwise backward inclusion of significant predictor variables for visit frequency and quality perception based on a sample of 1010 adult Norwegians. P-value significance levels: 0 ‘***’, 0.001 ’**’, 0.01 ’ *’, 0.05 ’ .’ , slopes and 95% confidence Intervals.*

| Variables |  | Perceived quality | | | Visit frequency | | | |
| --- | --- | --- | --- | --- | --- | --- | --- | --- |
|  |  | p | Slope | 97,5% CI | p | | Slope | 97.5% CI |
| Perceived quality | Good  Average  Bad |  |  |  | 0 (Ref)  0.075 .  0.024 * | | 0 (Ref)  -0.139  -0.319 | 0 (Ref)  (-0.293-0.014)  (-0.596- -0.414) |
| Frequency | Several times a week  Weekly  Less | 0 (Ref)  0.311  3.37e-05 *** | 0 (Ref)  -0.213  -0.17 | 0 (Ref)  (-0.203 -0.064)  (-0.441 – (-0.159)) | |  |  |  |
| Education | Lower  Higher |  |  |  | 0 (Ref)  0.003 ** | | 0 (Ref)  0.211 | 0 (Ref)  (0.074-0.349) |
| Region | Northern- and Central Norway  Oslo  Eastern Norway  Western- and Southern Norway | 0 (Ref)  0.298  0.004 **  0.065 . | 0 (Ref)  0.101  0.212  0.143 | 0 (Ref)  (-0.09 – 0.292)  (0.068 – 0.357)  (-0.009 – 0.295) |  | |  |  |
| Distance | <300m  300m – 5 km  > 5km | 0 (Ref)  <0.001 ***  0.112 | 0 (Ref)  -0.213  -0.17 | 0 (Ref)  (-0.330 – (-0.096)  (-0.38 – 0.038) | 0 (Ref)  6.36e-13 ***  < 2e-16 *** | | 0 (Ref)  -0.509  -1.068 | 0 (Ref)  (-0.646 - (-0.372))  (-1.316 – (-0.82)) |
| R^2^ (%) |  | 5.3 |  |  | 14.3 | |  |  |

Table 4: Linear regression model outcomes with stepwise backward inclusion of significant predictor variables for activities based on a sample of 1010 adult Norwegians. Presented as slope values and P-value significance levels: 0 ‘***’, 0.001 ’**’, 0.01 ’ *’, 0.05 ’ .’

|  | |  | Intrinsic | | | | | Extrinsic | | | Social | | | Active | | | Non-visitors | |
| --- | --- | --- | --- | --- | --- | --- | --- | --- | --- | --- | --- | --- | --- | --- | --- | --- | --- | --- |
|  |  | | Quietness | Get fresh air | | Relax | Experience Nature | Walk the dog | Food Collection | Play with children | visit/ take part in events | Meet friends | Picnic | Running | Cycling | Ball games | Passing | Do not visit |
|  | N | | 263 | | 509 | 288 | 397 | 153 | 121 | 189 | 81 | 175 | 89 | 145 | 140 | 43 | 497 | 74 |
| Perceived quality | Good | | 0 (Ref) | | 0 (Ref) | 0 (Ref) | 0 (Ref) |  | 0 (Ref) | - | - | - | - | 0 (Ref) | 0 (Ref) | - | - | 0 (Ref) |
|  | Average | | -0.398 * | | -0.728 *** | -0.356 . | -0.941 *** |  | -0.836 ** | - | - | - | - | -0.705 ** | 0.069 | - | - | 0.784 * |
|  | Bad | | -0.291 | | -0.796 ** | -0.796 * | -1.05 ** |  | -0.083 | - | - | - | - | 0.195 | - 1.376 . | - | - | 1.632 *** |
| Distance | < 300m | | - | | - | 0 (Ref) | 0 (Ref) | 0 (Ref) | - | 0 (Ref) | 0 (Ref) | - | - | 0 (Ref) | 0 (Ref) | 0 (Ref) | 0 (Ref) | - |
|  | 300m - 5km | | - | | - | -0.195 | -0.21 | -0.428 * | - | -0.447 * | -0.702 ** | - | - | -0.08 | 0.135 | -1.117 ** | 0.28 . | - |
|  | > 5km | | - | | - | -1 .003 ** | -0.931 ** | -0.671 . | - | -0.735 . | 0.026 | - | - | -1.849 * | -1.448 * | 0.268 | 0.39 | - |
| Gender | Female | | 0 (Ref) | | 0 (Ref) | 0 (Ref) | - | 0 (Ref) | 0 (Ref) | 0 (Ref) | - | - | 0 (Ref) | 0 (Ref) | 0 (Ref) | 0 (Ref) | 0 (Ref) | - |
|  | Male | | -0.345 * | | -0.33 *** | -0.328 * | - | -0.392 * | -0.784 *** | -0.532 ** | - | - | -0.418 . | 0.524 ** | 0.382 * | 1.538 *** | 0.442 ** | - |
| Age | 18-29 | | - | | 0 (Ref) | 0 (Ref) |  | 0 (Ref) | - | 0 (Ref) | 0 (Ref) | 0 (Ref) | 0 (Ref) | 0 (Ref) | - | 0 (Ref) | - | - |
|  | 30-39 | | - | | -0.181 | -0.167 | - | -0.088 | - | 1.442 *** | -0.772 * | -0.79** | 0.894 ** | -0.631 * | - | -2.135 *** | - | - |
|  | 40-49 | | - | | -0.427 . | -0.609 * | - | 0.275 | - | 0.503 | -1.239 ** | -1.601 *** | -0.215 | -0.973 ** | - | -0.907 . | - | - |
|  | 50-59 | | - | | -0.549 * | -0.69 * | - | 0.478 | - | 0.73 . | -1247 ** | -1.742 *** | -0.919 . | -1.137 ** | - | -2.145 ** | - | - |
|  | 60+ | | - | | -0.103 . | -0.555 * | - | -0.451 | - | 1.305 *** | -1.933 *** | -1.527 *** | -1.287 ** | -2.403 *** | - | - | - | - |
| Education | Lower | | - | | 0 (Ref) | - | 0 (Ref) | - | 0 (Ref) | - | - | - | - | 0 (Ref) | 0 (Ref) | 0 (Ref) | 0 (Ref) | - |
|  | Higher | | - | | 0.256 . | - | 0.22 | - | 0.697 ** | - | - | - | - | 0.582 * | 0.405 . | -0.698 | -0.345 * | - |
| Children <18 | None | | 0 (Ref) | | - | - | - | - | - | 0 (Ref) | 0 (Ref) | - | - | - | - | 0 (Ref) | 0 (Ref) | - |
|  | One | | -0.349 * | | - | - | - | - | - | 2.294 *** | 0.482 . | - | - | - | - | 1.197 ** | -0.44 ** | - |
| Degree of urbanisation | Rural | | 0 (Ref) | | - | 0 (Ref) | 0 (Ref) | - | 0 (Ref) | - | - | - | - | 0 (Ref) | - | - | 0 (Ref) | 0 (Ref) |
|  | Suburban | | -0.526 ** | | - | -0.179 | -0.56 5** | - | -0.663 ** | - | - | - | - | 0.636 * | - | - | 0.449 * | -1.087 ** |
|  | Urban | | 0.124 | | - | 0.514 ** | -0.597 *** | - | -1.1 *** | - | - | - | - | 0.777 ** | - | - | 0.926 *** | -1.394 ** |
